# Construction of chromosome segment substitution lines enables QTL mapping for flowering and morphological traits in *Brassica rapa*

**DOI:** 10.3389/fpls.2015.00432

**Published:** 2015-06-09

**Authors:** Xiaonan Li, Wenke Wang, Zhe Wang, Kangning Li, Yong Pyo Lim, Zhongyun Piao

**Affiliations:** ^1^Department of Horticulture, Shenyang Agricultural UniversityShenyang, China; ^2^Molecular Genetics and Genomics Lab, Department of Horticulture, Chungnam National UniversityDaejeon, South Korea

**Keywords:** *Brassica rapa*, chromosome segment substitution lines (CSSLs), quantitative trait loci (QTL), morphological traits, marker-assisted selection (MAS)

## Abstract

Chromosome segment substitution lines (CSSLs) represent a powerful method for precise quantitative trait loci (QTL) detection of complex agronomical traits in plants. In this study, we used a marker-assisted backcrossing strategy to develop a population consisting of 63 CSSLs, derived from backcrossing of the F_1_ generated from a cross between two *Brassica rapa* subspecies: “Chiifu” (ssp. *pekinensis*), the *Brassica* “A” genome-represented line used as the donor, and “49caixin” (ssp. *parachinensis*), a non-heading cultivar used as the recipient. The 63 CSSLs covered 87.95% of the *B. rapa* genome. Among them, 39 lines carried a single segment; 15 lines, two segments; and nine lines, three or more segments of the donor parent chromosomes. To verify the potential advantage of these CSSL lines, we used them to locate QTL for six morphology-related traits. A total of 58 QTL were located on eight chromosomes for all six traits: 17 for flowering time, 14 each for bolting time and plant height, six for plant diameter, two for leaf width, and five for flowering stalk diameter. Co-localized QTL were mainly distributed on eight genomic regions in A01, A02, A05, A06, A08, A09, and A10, present in the corresponding CSSLs. Moreover, new chromosomal fragments that harbored QTL were identified using the findings of previous studies. The CSSL population constructed in our study paves the way for fine mapping and cloning of candidate genes involved in late bolting, flowering, and plant architecture-related traits in *B. rapa*. Furthermore, it has great potential for future marker-aided gene/QTL pyramiding of other interesting traits in *B. rapa* breeding.

## Introduction

Segregating populations have been widely used in genetic mapping, quantitative trait loci (QTL) analysis, and gene discovery. An appropriate mapping population can facilitate the identification of functional loci. Primary mapping populations such as F_2_, backcross populations (BC_1_), recombinant inbred lines (RILs), and doubled haploids (DHs) have been widely used for genetic map construction (Kole et al., 1997; Choi et al., 2007; Li et al., 2010; Ge et al., 2011a) and QTL/gene identification (Muangprom and Osborn, 2004; Lou et al., 2007; Li et al., 2009, 2013) in *Brassica*. However, several disadvantages of these population types, such as the short-lived and temporary nature of F_2_/BC_1_ and their bias toward QTL mapping of relatively large-effect loci (Teutonico and Osborn, 1995; Lou et al., 2007; Rahman et al., 2007), have seriously limited detailed genetic analyses such as QTL fine mapping and cloning.

Advanced mapping populations, including chromosome segment substitution lines (CSSLs), recombinant chromosome substitution lines (RCSLs), introgression lines (ILs), backcross inbred lines (BILs), and near-isogenic lines (NILs), allow to achieve precise QTL identification. These mapping populations have been used for many species such as rice (Ebitani et al., 2005; Tian et al., 2006; Gu et al., 2012), tomato (Eshed and Zamir, 1995; Monforte and Tanksley, 2000), lettuce (Jeuken and Lindhout, 2004), and wheat (Miura et al., 2002; Liu et al., 2006).

CSSLs are a series of ILs produced by crossing and backcrossing the donor and recipient parents by using marker-assisted selection (MAS), that finally contain the entire genome information of the donor parent. Each CSSL carries one or more donor chromosome segments in the genetic background of the recurrent parent, thereby eliminating genetic background noise and allowing the detection of QTL with additive minor effects that are always masked in F_2_ or RIL primary populations (Yamamoto et al., 2009). In addition, development of CSSLs by using MAS has provided a new breeding strategy for the improvement of cultivated species and allowing the introgression of novel genes or alleles from wild relatives. This strategy has been successfully applied in various crops (Shim et al., 2010; Fonceka et al., 2012; Wang et al., 2013; Furuta et al., 2014). Furthermore, the use of these CSSLs has enabled the accurate identification of QTL that govern complex agronomic (Lin et al., 2002; Wang et al., 2006) and quality traits (Wan et al., 2004), as well as allowed the detection of the interaction between QTL and multi-environments (Wan et al., 2005).

*Brassica rapa* is an important species among the six economically cultivated *Brassica* species of U's triangle (UN, 1935), consumed worldwide. The long and worldwide cultivation history and ongoing breeding have resulted in various morphotypes within this species, such as leafy vegetables, oilseeds, and fodder. Until recently, few genetic studies had been performed using *B. rapa* species despite the existence of a wide variation in morphological traits in this species. A study of the genes underlying *B. rapa* morphological traits was performed by Song et al. (1995) by using an F_2_ population. Lou et al. (2007) performed the genetic dissection of 20 morphological traits by using multiple populations (DH, RIL, F_2:3_, and BC_1_) derived from parental lines that involved three main *B. rapa* morphotypes (oilseed, leafy, and turnip types). Recently, several QTL regions containing candidate genes for flowering time and leaf morphological traits have been identified by Li et al. (2009) and Li et al. (2013) by using F_2:3_ populations derived from leafy and early-flowering oilseed types of *B. rapa*. Even though many QTL have been detected in these populations, to some extent, the use of primary mapping populations has limited the progress of fine mapping and QTL pyramiding of complex morphological traits in *B. rapa*. Therefore, development of advanced mapping populations such as CSSLs is required for precise QTL identification in *B. rapa*. This would enhance the comprehensive understanding of complex quantitative traits and pave the way for subsequent gene discovery.

In this study, we described the development of a novel CSSL population by using MAS with the heading Chinese cabbage “Chiifu,” a model plant for *Brassica* A genome sequencing, and the early-flowering stalk-type inbred line “49caixin.” Furthermore, QTL for six morphological traits were identified using these CSSLs in order to demonstrate their potential use in *B. rapa* species.

## Materials and methods

### Plant materials

**Figure 1:**
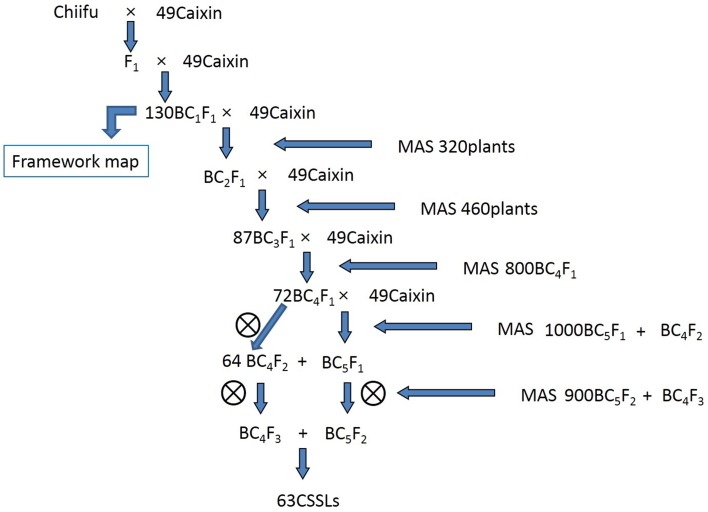
**Breeding scheme for constructing the chromosome segment substitution line (CSSL) population by using “Chiifu” as the donor parent in the genetic background of “49caixin.”**.

In this study, two *B. rapa* lines, “Chiifu” and “49caixin,” were used to develop CSSLs. The heading Chinese cabbage inbred line “Chiifu” (*B. rapa* ssp. *pekinensis*), used for *Brassica* A genome sequencing by the Multinational *Brassica* Genome Project, was used as the donor parent. The inbred line “49caixin” (*B. rapa* ssp. *parachinensis*), a non-heading and early-flowering type, was used as the recurrent parent. The F_1_ plant was crossed with the recurrent parent “49caixin” to produce BC_1_F_1_ individuals. BC_4_F_2_ and BC_5_F_2_ populations were produced by four and five rounds of backcrossing, respectively, that is, by crossing BC individuals with “49caixin” by using MAS, followed by self-fertilization in one generation (Figure 1). All crosses were performed in a greenhouse at the Shenyang Agricultural University.

### Simple sequence repeat marker analysis

A high-density linkage map based on unigene-derived microsatellite markers (UGMS) has been developed previously (Wang et al., 2014). The physical position of 251 mapped markers was determined by anchoring the marker sequences to the *B. rapa* reference genome by using BLAST analysis (http://brassicadb.org/brad/). On the basis of the positions of the markers, their amplification efficiency and clearly identifiable polymorphisms, a total of 110 markers consisting of UGMS markers (designated as “sau_um”) and public simple sequence repeat (SSR) markers (prefixed by “cnu,” ”nia,” “BRMS,” and “mENA”), evenly distributed throughout the *B. rapa* genome were selected for framework map construction and screening of individuals in each backcross generation.

Young leaf tissue was collected from the two parental lines and each BC individual and maintained at −80°C for DNA isolation. DNA was extracted according to the CTAB method (Rogers and Bendich, 1988) with minor modifications. The DNA concentration was determined using a nucleic acid detector and diluted to 5 ng·μl^−1^ for PCR. PCR and amplification conditions for genotyping of individuals according to UGMS and public SSR markers have been described by Li et al. (2010) and Ge et al. (2011a), respectively.

### Substituted chromosome segment length estimation in CSSLs

**Figure 2:**
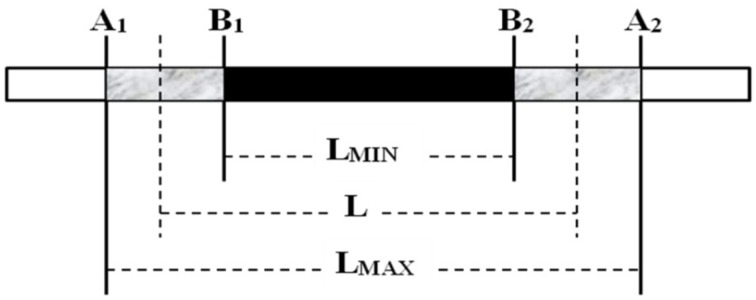
**Determination of the lengths of the substituted segments in the CSSLs**.

Substituted chromosome segment length estimation in CSSLs was performed using the “graphical genotype” method reported by Young and Tanksley (1989) (Figure 2). The letter A is used to represent the recurrent parental genotype and B, the donor genotype. The letter L stands for estimated length of CSSL, Lmax for maximum length, and Lmin for minimum length. A chromosome segment flanked by two molecular markers of the donor genotype (BB) was considered as 100% donor type, a chromosome segment flanked by two markers of the recurrent parental genotype (AA) was considered as 0% donor type, and a chromosome segment flanked by one marker of the donor genotype and one of the recurrent parental genotype (AB) was considered as 50% donor type and calculated using the following formula: L = (L_max_ + L_min_)/2 (Figure 2).

### Phenotyping for morphological and flowering traits in CSSLs

For phenotypic investigation, the 63 CSSLs and the two parental lines were grown at an experimental field in Shenyang Agricultural University, Shenyang, China, in August 2013. The plants were arranged in a randomized block design with three replicates and 12 plants per line in each of the three blocks. The spacing between plants and rows was 20 and 30 cm, respectively. A total of six morphology-related traits were measured, namely, bolting time, flowering time, plant height, plant diameter, leaf width, and flowering stalk diameter. Days to bolting (DB) was defined as the number of days from seed sowing to the emergence of a 5-cm stalk. Days to flowering (DF) was defined as the number of days from seed sowing to opening of the first flower. Plant height (PH) was measured from the ground to the top of the stem on the day the first flower blossomed. Plant diameter (PD) represented the maximum width of the vertical projection of the basal leaves. Leaf width (LW) and flowering stalk diameter (FSD) were measured at the widest point of the biggest leaf and of the main flowering stalk, respectively. Eighteen CSSLs showing significantly different values for DB and DF from the recurrent parent line, “49caixin,” according to Dunnett's *t*-test (Dunnett, 1985) were grown again in January 2013 to verify the QTL for DB and DF. The SAS 9.0 program (SAS Institute, Inc., Cary, NC, USA) was used for correlation coefficient analysis between two traits.

### CSSL-based QTL analysis

QTL analysis was performed on CSSLs that showed a significantly different trait value compared to that of the recurrent parent “49caixin” on the basis of Dunnett's *t*-test (Dunnett, 1985) at a probability level of 0.001 (*P* < 0.001) and assigned to the chromosome regions of these CSSLs. When a QTL was detected in multiple CSSLs, it was considered to be located on overlapping chromosome segments among these CSSLs. A QTL was considered to be on non-overlapping chromosome segments, when it was detected in only one CSSL. The additive effect of QTL was calculated according to the method described by Eshed and Zamir (1995). The additive effect was half of the phenotypic difference between each CSSL and “49caixin,” and the additive effect contribution was calculated from the additive effect value divided by the phenotype value of “49caixin.”

## Results

### CSSL population development and MAS

A framework map was constructed on the basis of the BC_1_F_1_ generation by using the 110 genome-wide distributed SSR markers. The markers were evenly distributed among the 10 *B. rapa* chromosomes, with an average interval of 2.33 Mb between each pair of markers (Table 1).

**Table 1:** **Summary of the markers used to develop the CSSLs**.

**Chromosome**	**Number of markers**	**Average distance (Mb)**	**Maximum distance between near markers (Mb)**
A01	8	2.04	8.76
A02	14	1.86	5.37
A03	17	1.67	3.22
A04	4	3.16	5.77
A05	12	1.84	4.50
A06	10	2.63	3.65
A07	9	2.05	4.04
A08	11	1.80	3.91
A09	16	2.06	4.73
A10	9	1.96	3.16
Total	110	2.33	–

Whole-genome surveys were performed using MAS. The 110 SSR markers of the framework map were used to genotype a total of 130, 320, and 460 individuals from BC_1_F_1_, BC_2_F_1_, and BC_3_F_1_ populations, respectively (Figure 1). From each backcross generation, individuals were selected using the CSSL-finder software (http://mapdisto.free.fr/CSSLFinder/). A greedy algorithm is used for line selection. The optimal line was selected for the segment covering the first marker in chromosome A01, and then along each chromosome until all markers are covered. Foreground selection of populations was conducted using the two SSR markers defining the introgressed segments and background selection was performed using genome-wide SSR markers defining the recipient genome recovery in each round of backcross. An optimal line is the one has minimal number of donor segments. All the selected lines ensured the nearly coverage of whole genome of the donor parent. Using both foreground and background selection, 87 BC_3_F_1_ individuals were selected and backcrossed with “49caixin” to generate BC_4_F_1_. In spring 2012, 800 BC_4_F_1_ individuals were obtained and genotyped. We selected 72 plants from the 800 BC_4_F_1_ individuals for self-fertilization in order to produce BC_4_F_2_ and for backcrossing to produce BC_5_F_1_. Subsequently, 64 of 1000 individuals from the BC_4_F_2_ and BC_5_F_1_ populations that had less than five homozygous substituted segments from the donor “Chiifu” were selected for self-fertilization to produce BC_4_F_3_ and BC_5_F_2_, respectively. Finally, in spring 2013, 63 out of 900 BC_4_F_3_ and BC_5_F_2_ individuals that carried homozygous target chromosome segments were obtained by MAS as our final CSSL population (Figure 1).

### Distribution of substitution segments on chromosomes in CSSLs

**Figure 3:**
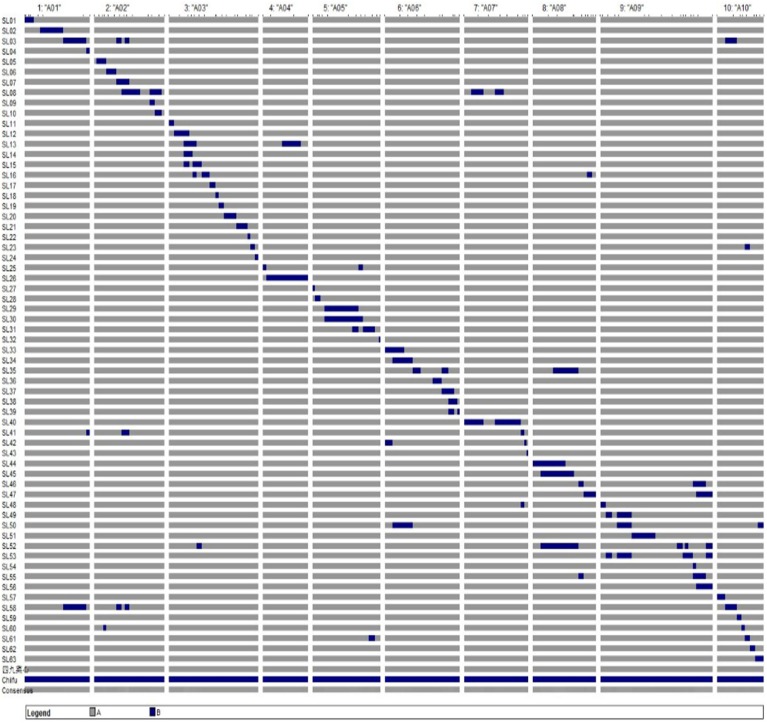
**Graphical genotypes of the 63 CSSLs**. Gray represents the genotype of the recurrent parent “49caixin.” Black represents the genotype of the donor parent “Chiifu.”

The combination of crosses and backcrosses with MAS allowed the development of a set of 63 CSSLs with 102 substituted chromosome segments from the donor “Chiifu” in the genetic background of “49caixin.” The distribution of the segments along the chromosomes ranged from three on A04 to 17 on A03 and A09 (Table 2). Graphical genotypes of the 63 CSSLs determined using 110 SSR markers distributed across 10 chromosomes are shown in Figure 3. Among the 63 CSSLs, 39 lines (61.9% of the total lines) carried one unique donor parental substitution segment, 15 lines carried two substitution segments, and nine lines carried more than three donor substitution segments (Figure 3). The length of the substitution chromosome segments in the 63 CSSLs ranged from 0.05 Mb on A07 to 17.18 Mb on A04, with an average of 3.69 Mb (Figure 3).

**Table 2:** **Chromosome coverage of substituted segments in the CSSLs**.

**Chromosome**	**Total length of *B. rapa* chromosome (Mb)^a^**	**Number of substituted segments**	**Total substituted segments length (Mb)^b^**	**Target substituted segments length (Mb)^c^**	**Percentage of chromosome coverage (%)**
A01	28.61	6	42.87	26.60	92.97
A02	27.85	13	29.25	20.02	62.68
A03	31.72	17	42.52	37.76	100.00
A04	18.97	3	24.97	18.97	100.00
A05	23.94	9	37.49	32.05	89.10
A06	26.27	11	41.06	27.03	86.12
A07	22.59	8	27.21	19.08	84.44
A08	21.60	8	52.06	29.11	100.00
A09	37.12	17	53.45	32.75	77.00
A10	17.60	10	25.06	17.59	100.00
Total	256.27	102	375.94	269.61	89.23 (average)

a *The total length of B. rapa genome was referred to as Wang et al. (2011)*.

b *All substituted segments introgressed from “Chiifu” into “49caixin” genome*.

c *Substituted segments from the target chromosome of donor “Chiifu” genome, excluding residual non-targeted fragment of donor*.

### Genome coverage of substitution segments in the CSSL population

All SSR markers on the linkage map were anchored to the *B. rapa* reference genome by using BLAST analysis (http://brassicadb.org/brad/), and then their physical positions were determined. The total length of the substitution segments in the population was 375.94 Mb, that is, 1.47 times the reference *B. rapa* genome (Wang et al., 2011), and it from 24.97 Mb on A04 to 53.45 Mb on A09. The total coverage of the substitution chromosome segments of the *B. rapa* genome was 269.61 Mb. The average coverage rate of substitution segments per chromosome was 87.95% and ranged from 62% on A02 to 100% on A03, A04, A08, and A10 (Table 2).

The genome of the donor parent “Chiifu” was introgressed in the CSSLs in contiguous or overlapping segments on chromosomes A03, A04, A08, and A10. In contrast, as shown by the gaps, it was not successfully introgressed in the CSSLs on chromosomes A01 (2.05 Mb between sau_um365 and sau_um367), A02 (2.50 Mb between cnu-295 and sau_um142), A05 (1.20 Mb between cnu_425 and sau_um517), A06 (2.45 Mb between BnGMS288 and mENASa), A07 (4.05 Mb between cnu_062 and sau_um639), and A09 (6.85 Mb between sau_um138 and cnu_581) (Figure 3).

### Variations in phenotypic traits

**Figure 4:**
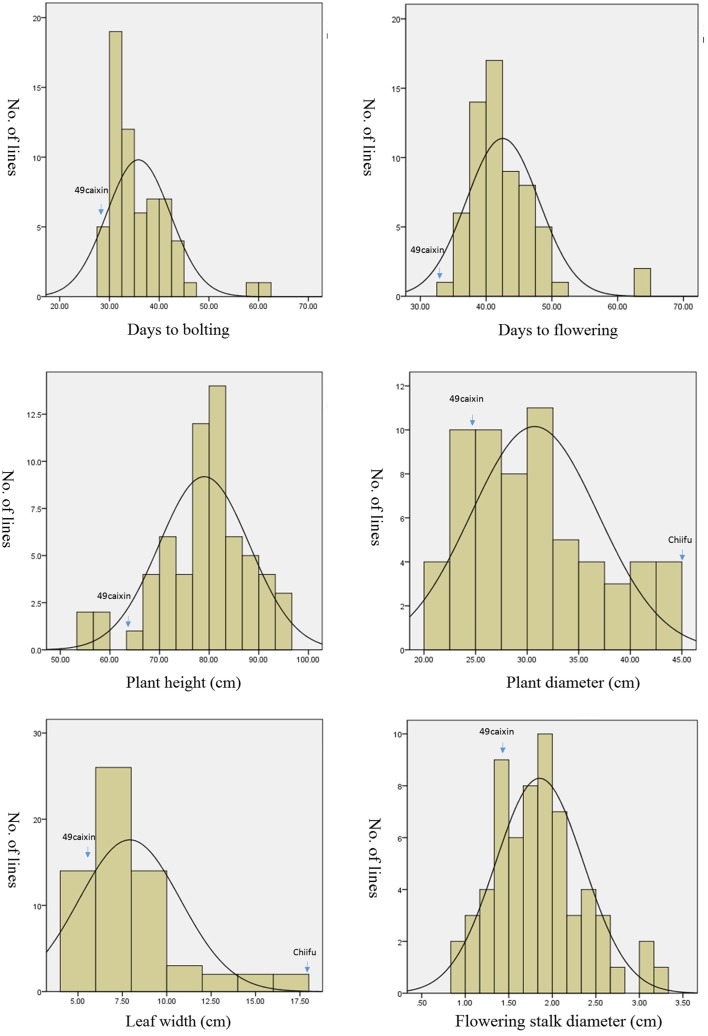
**Phenotypic distribution of six morphological traits in the 63 CSSLs**. The phenotypic value of two parental lines for each trait is pointed. Chiifu did not flower until seed harvest time. No phenotypic value of “Chiifu” is reported for DB, DF, PH, and FSD.

Phenotypic variation between the CSSL population and the two parental lines was observed for six morphology-related traits (Table 3). The two parental lines also showed significant differences. The recurrent parent “49caixin” is an early-flowering type *B. rapa*, whereas the donor parent “Chiifu” is a heading-type Chinese cabbage that did not flower until seed harvest time. In addition, significant differences were observed in LW between the parents. Distribution analysis of the phenotypic values of the six traits showed a continuous normal distribution in the CSSL population, suggesting that each trait was governed by multiple genes (Figure 4). All traits were significantly correlated in the CSSLs, except PH, which was not correlated with either DB or DF (Table 4). A highly significant positive correlation was observed between DB and DF (*r* = 0.955), LW and FSD (*r* = 0.746), PD and FSD (*r* = 0.739), and LW and PD (*r* = 0.717), whereas the correlation between PH and LW (*r* = 0.294) and PH and PD (*r* = 0.336) was comparatively low (Table 4).

**Table 3:** **Phenotypic distribution for six morphological traits in the CSSL population**.

**Traits**	**Parents value**		**Distribution in the CSSL population**					
	**49caixin**	**Chiifu^a^**	**Max**	**Min**	**Mean**	**Standard deviation**	**Kurtosis**	**Skewness**
DB	28.20	−	64.00	26.00	35.58	0.192	2.459	1.425
DF	32.45	−	67.00	30.00	42.40	0.175	2.291	0.978
PH	63.10	−	96.33	54.67	79.01	0.567	2.387	−0.622
PD	24.67	47.10	43.50	20.67	30.72	0.366	−0.048	0.500
LW	5.88	19.80	17.37	4.20	7.91	0.147	2.134	1.331
FSD	1.30	−	3.20	0.97	1.85	0.305	1.325	0.883

a *“Chiifu” did not flower until the populations seed harvest*.

**Table 4:** **Phenotypic correlations among six morphological traits in the CSSL population**.

**Traits**	**FSD**	**DB**	**DF**	**PH**	**PD**
DB	0.638^**^				
DF	0.618^**^	0.955^**^			
PH	0.466^**^	0.089	0.119		
PD	0.739^**^	0.679^**^	0.665^**^	0.336^**^	
LW	0.746^**^	0.627^**^	0.641^**^	0.294^**^	0.717^**^

** *indicates the significant level at 1%*.

### QTL mapping of morphological and flowering traits in the CSSL population

**Figure 5:**
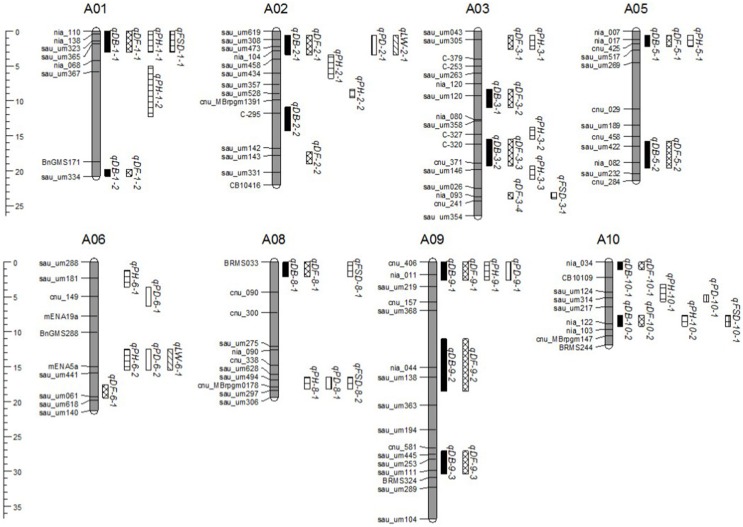
**QTL distribution on eight *Brassica rapa* chromosomes detected in the CSSL population**. QTL names on the right of each rectangle are indicated by abbreviations of trait names as shown in Table 5.

QTL were detected using a *t*-test based on the difference between the mean of each CSSL and “49caixin” for the six morphological traits. A total of 58 QTL for all traits were located on eight chromosomes: 17 for DF, 14 each for DB and PH, 6 for PD, 2 for LW, and 5 FSD (Table 5, Figure 5). Almost all the trait-enhancing alleles were derived from the donor parent “Chiifu.”

**Table 5:** **QTL location of morphological traits in the CSSL population**.

**Traits**	**CSSL**	**Marker interval**	**QTL Designation**	**Linkage group**	***P*-value**	**Mean ± SE**	**Additive value**	**Additive effect (%)**
DB	49caixin					28.20±0.12		
	Chiifu					_		
	SL01	nia_110~sau_um365	*qDB-1-1*	A01	8.20E-05^***^	37.28±0.54	4.54	16.09
	SL04	sau_um334	*qDB-1-2*	A01	7.20E-06^***^	40.89±0.40	6.34	22.49
	SL05	sau_um308~nia_104	*qDB-2-1*	A02	8.18E-07^***^	60.79±0.61	16.29	57.77
	SL08	cnu_295	*qDB-2-2*	A02	1.72E-04^***^	33.67±0.38	2.73	9.69
	SL16	sau_um120	*qDB-3-1*	A03	5.26E-05^***^	39.00±0.58	5.40	19.14
	SL20	cnu_320~cnu_371	*qDB-3-2*	A03	3.08E-05^***^	40.72±0.59	6.26	22.20
	SL28	nia_017~cnu_425	*qDB-5-1*	A05	9.55E-05^***^	34.77±0.40	3.29	11.65
	SL31	sau_um422~nia_082	*qDB-5-2*	A05	5.26E-05^***^	39.00±0.58	5.40	19.14
	SL44	BRMS033	*qDB-8-1*	A08	2.12E-05^***^	41.29±0.56	6.54	23.19
	SL48	cnu_406~nia_011	*qDB-9-1*	A09	7.04E-05^***^	43.33±0.88	7.57	26.82
	SL51	nia_044~sau_um138	*qDB-9-2*	A09	1.80E-06^***^	40.62±0.26	6.21	22.01
	SL53	sau_um445~sau_um111	*qDB-9-3*	A09	1.26E-08^***^	58.83±0.17	15.32	54.30
	SL57	nia_034	*qDB-10-1*	A10	6.73E-06^***^	44.00±0.50	7.90	28.00
	SL62	nia_112	*qDB-10-2*	A10	3.04E-04^***^	40.00±1.00	5.90	20.91
DF	49caixin					32.45±0.22		
	Chiifu					_		
	SL01	nia_110~sau_um365	*qDF-1-1*	A01	1.82E-05^***^	44.71±0.46	6.13	18.88
	SL04	sau_um334	*qDF-1-2*	A01	6.64E-06^***^	45.21±0.35	6.38	19.65
	SL05	sau_um308~nia_104	*qDF-2-1*	A02	1.31E-07^***^	64.87±0.33	16.21	49.95
	SL09	sau_um143	*qDF-2-2*	A02	6.88E-04^***^	40.47±0.82	4.01	12.35
	SL12	sau_um305	*qDF-3-1*	A03	4.52E-04^***^	41.30±0.81	4.43	13.63
	SL16	sau_um120	*qDF-3-2*	A03	1.55E-05^***^	42.33±0.33	4.94	15.22
	SL20	cnu_320~cnu_371	*qDF-3-3*	A03	4.91E-07^***^	46.65±0.10	7.09	21.87
	SL22	nia_093	*qDF-3-4*	A03	3.82E-05^***^	40.33±0.33	3.94	12.14
	SL28	nia_017~cnu_425	*qDF-5-1*	A05	5.60E-06^***^	41.35±0.17	4.45	13.70
	SL31	sau_um422~nia_082	*qDF-5-2*	A05	6.68E-06^***^	44.67±0.33	6.12	18.82
	SL38	sau_um061	*qDF-6-1*	A06	4.64E-04^***^	40.67±0.75	4.12	12.65
	SL44	BRMS033	*qDF-8-1*	A08	1.09E-06^***^	45.79±0.17	6.67	20.55
	SL48	cnu_406~nia_011	*qDF-9-1*	A09	1.91E-05^***^	47.00±0.58	7.27	22.41
	SL51	nia_044~sau_um138	*qDF-9-2*	A09	1.31E-05^***^	46.12±0.48	6.83	21.05
	SL53	sau_um445~sau_um111	*qDF-9-3*	A09	9.91E-08^***^	62.70±0.27	15.12	46.60
	SL57	nia_034	*qDF-10-1*	A10	1.02E-05^***^	49.50±0.58	8.52	26.26
	SL62	nia_122	*qDF-10-2*	A10	3.42E-04^***^	46.33±1.20	6.94	21.38
PH	49caixin					63.10±0.80		
	Chiifu					_		
	SL01	nia_110~sau_um365	*qPH-1-1*	A01	5.53E-04^***^	87.67±2.31	12.28	19.47
	SL02	sau_um367	*qPH-1-2*	A01	8.52E-04^***^	90.00±2.89	13.45	21.32
	SL06	sau_um458~sau_um434	*qPH-2-1*	A02	1.00E-04^***^	79.33±0.67	8.12	12.86
	SL08	sau_um528	*qPH-2-2*	A02	1.43E-04^***^	83.33±1.17	10.12	16.03
	SL12	sau_um305	*qPH-3-1*	A03	6.09E-04^***^	83.25±1.89	10.08	15.97
	SL19	cnu_327	*qPH-3-2*	A03	9.85E-04^***^	89.67±2.97	13.28	21.05
	SL21	sau_um146~sau_um026	*qPH-3-3*	A03	1.89E-05^***^	83.42±0.30	10.16	16.10
	SL28	nia_107~cnu_425	*qPH-5-1*	A05	2.30E-04^***^	84.83±1.53	10.87	17.22
	SL33	sau_um181	*qPH-6-1*	A06	1.16E-04^***^	76.81±0.44	6.85	10.86
	SL36	mENA5a	*qPH-6-2*	A06	3.94E-05^***^	86.83±0.90	11.87	18.81
	SL47	sau_um494~cnu_MBrpgm0178	*qPH-8-1*	A08	1.33E-04^***^	91.77±1.81	14.33	22.72
	SL48	cnu_406~nia_011	*qPH-9-1*	A09	1.24E-04^***^	82.00±1.00	9.45	14.98
	SL60	sau_um124~sau_um314	*qPH-10-1*	A10	8.30E-04^***^	83.67±2.13	10.28	16.30
	SL62	nia_122	*qPH-10-2*	A10	1.76E-04^***^	96.33±2.33	16.62	26.33
PD	49caixin					24.67±0.30		
	Chiifu					47.10±3.73		
	SL05	sau_um308~nia_104	*qPD-2-1*	A02	5.32E-05^***^	43.14±0.97	9.24	37.44
	SL34	cnu_149	*qPD-6-1*	A06	5.65E-04^***^	21.30±0.15	−1.68	−6.82
	SL36	mENA5a	*qPD-6-2*	A06	3.62E-04^***^	33.20±0.70	4.27	17.30
	SL47	sau_um494~cnu_MBrpgm0178	*qPD-8-1*	A08	6.52E-05^***^	40.10±0.84	7.72	31.28
	SL48	cnu_046~nia_011	*qPD-9-1*	A09	3.32E-04^***^	35.33±0.88	5.33	21.62
	SL60	sau_um314	*qPD-10-1*	A10	3.29E-04^***^	39.79±1.28	7.56	30.66
LW	49caixin					5.88±0.44		
	Chiifu					19.80±0.33		
	SL05	sau_um308~nia_104	*qLW-2-1*	A02	1.53E-04^***^	16.18±0.59	5.15	87.71
	SL36	mENA5a	*qLW-6-1*	A06	7.33E-05^***^	17.37±0.52	5.75	97.80
FSD	49caixin					1.30±0.06		
	Chiifu					_		
	SL01	nia_110~sau_um365	*qFSD-1-1*	A01	1.15E-04^***^	2.52±0.06	0.67	46.64
	SL22	nia_093	*qFSD-3-1*	A03	6.32E-04^***^	1.90±0.02	0.30	22.98
	SL44	BRMS033	*qFSD-8-1*	A08	2.71E-04^***^	2.14±0.04	0.42	32.11
	SL47	sau_um494~cnu_MBrpgm0178	*qFSD-8-2*	A08	6.54E-04^***^	1.90±0.02	0.30	23.11
	SL62	nia_112	*qFSD-10-1*	A10	9.13E-05^***^	2.35±0.03	0.13	40.40

*** *indicates the significant level at 0.1%*.

#### Days to bolting (DB)

The DB value was 28.20 ± 0.12 days for “49caixin.” It ranged from 26 to 64 days among the 63 CSSLs (Table 3). Significant differences in the DB values were detected between 14 CSSLs and “49caixin” (Table 5). The 14 QTL were distributed on seven chromosomes: two QTL each on A01, A02, A03, A05, and A10; three on A09; and one on A08 (Figure 5). In all the 14 lines, donor “Chiifu” alleles at the DB QTL conferred a late-bolting phenotype, with positive additive values in the range of 2.73–16.29. The additive effects ranged from 9.69 to 57.77% among the 14 QTL. The QTL *qDB-2-1* on A02 and *qDB-9-3* on A09, identified in lines SL05 and SL53, were the two major QTL that showed the highest additive values (16.29 and 15.32, respectively; Table 5).

The 14 QTL carried by each CSSL were used for further QTL verification in the winter season. Four QTL, *qDB-2-1, qDB-3-2, qDB-8-1*, and *qDB-9-3*, carried by SL05, SL16, SL44, and SL53, respectively, were confirmed. The additive values for these QTL were 3.35, 1.24, 3, and 5.9, respectively, with additive effects of 9.87, 3.64, 8.86, and 17.4%, respectively.

#### Days to flowering (DF)

The DF value was 32.45 ± 0.22 days for “49caixin.” It ranged from 30 to 67 days among the 63 CSSLs (Table 3, Figure 4). A total of 17 lines had a significantly different DF value compared to “49caixi,” carrying 17 QTL genomic regions. These 17 QTL were mapped to eight chromosomes: A03 (four QTL); A09 (three QTL); A01, A02, A05, and A10 (two QTL each); and A06 (one QTL) (Figure 4). In all these 17 lines, donor “Chiifu” alleles at the DF QTL conferred a late-flowering phenotype, with positive additive values in the range of 3.94–16.21. The additive effects varied from 12.14 to 49.95%. The QTL *qDF-2-1* on A02 and *qDF-9-3* on A09 from lines SL05 and SL53 had the highest additive values (16.21 and 15.12, respectively) (Table 5), suggesting that these two genomic regions harbored major genes responsible for flowering time.

The 17 QTL carried by each CSSL were used for further QTL verification in the winter season. Three QTL, *qDF-2-1, qDF-8-1*, and *qDF-9-3*, carried by SL05, SL44, and SL53, respectively, were detected; the additive values for these QTL were 4.93, 2.82, and 7.56, respectively, with additive effects of 10.89, 6.22, and 16.68%, respectively.

#### Plant height (PH)

The PH value was 63.10 ± 0.80 cm for “49caixin.” It ranged from 54.67 to 96.33 cm among the 63 CSSLs (Table 3, Figure 4). A total of 14 PH QTL were detected by comparing the 63 CSSLs and “49caixin” (Table 5). Of these, three QTL were detected on A03; two each on A01, A02, A06, and A10; and one each on A05, A08, and A09 (Figure 5). At all QTL, the donor alleles were involved in increasing PH with positive additive values in the range of 6.85–16.62. The positive allele with the highest additive effect value (16.62) and highest additive effect contribution (26.33%) was derived from line SL62 at QTL *qPH-10-2* (Table 5).

#### Plant diameter (PD)

The PD value was 24.67 cm and 47.10 cm for “49caixin” and “Chiifu.” It ranged from 20.67 to 43.50 cm among the CSSLs (Table 3, Figure 4). The comparison between the 63 CSSLs and “49caixin” allowed the identification of six lines carrying QTL for PD. Six QTL were located on five chromosomes: two on A06; and one each on A02, A08, A09, and A10. In lines SL05, SL36, SL47, SL48, and SL60, the presence of donor alleles at the PD QTL increased PD with a positive additive value (4.27–9.24), and additive effect contribution (17.30–37.44%), while in the line SL34, one QTL (*qPD-6-1*) contributed to the decrease in PD with a negative additive value (−1.68) and additive effect (−6.82%) (Table 5).

#### Leaf width (LW)

The LW value was 5.88 ± 0.44 cm for “49caixin.” It ranged from 4.20 to 17.37 cm among the 63 CSSLs (Table 3, Figure 4). Two CSSLs showed significantly different LW compared to “49caixin.” QTL *qLW-2-1* was on A02 and had an additive effect value of 5.15 and additive effect contribution of 87.71%. QTL *qLW-6-1* was on A06 and had an additive effect value of 5.75 and additive effect contribution of 97.8% (Table 5, Figure 4).

#### Flowering stalk diameter (FSD)

The FSD value was 1.30 ± 0.06 cm for “49caixin.” It ranged from 0.97 to 3.20 cm among the individuals of the CSSL population (Table 3, Figure 4). A total of five lines carrying five genomic regions had an FSD significantly different from “49caixin.” Five QTL were identified in four chromosomes: two on A08; and one each on A01, A03, and A10 (Table 5, Figure 5). The donor alleles at all QTL were associated with increased FSD, with a positive additive effect value that ranged from 0.13 to 0.67. The additive effect contribution ranged from 22.98% at QTL *qFSD-3-1* to 46.64% at QTL *qFSD-1-1*(Table 5).

### QTL cluster regions

A total of eight genomic regions harboring QTL controlling three or more morphological traits were identified: four QTL (*qDB-1-1, qDF-1-1, qPH-1-1*, and *qFSD-1-1*) were co-localized at the top of A01 (0.1–1.8 Mb); four QTL controlling DB, DF, PD, and LW (*qDB-2-1, qDF-2-1, qPD-2-1*, and *qLW-2-1*) were located at the top of A02 (1.4–4.2 Mb); three QTL (*qDB-5-1, qDF-5-1*, and *qPH-5-1*) were located at the top of A05 (2.5–3.2 Mb); the middle to bottom of A06 (13.7–18.6 Mb) contained three QTL for PH, PD, and LW; the top of A08 (1.9–3.1 Mb) harbored three QTL controlling DB, DF, and FSD; the bottom of A08 (18.8–19.8 Mb) also contained three QTL for PH, PD, and FSD; four QTL (*qDB-9-1, qDF-9-1, qPH-9-1*, and *qPD-9-1*) were co-localized at the top of A09 (0.0–1.8 Mb); and, finally, four QTL (*qDB-10-2, qDF-10-2, qPH-10-2*, and *qFSD-10-1*) were co-localized at the bottom of A10 (12.9–13.7 Mb) (Figure 5).

## Discussion

In *B. rapa*, although numerous QTL-mapping studies have been conducted for identifying agronomical traits (Ge et al., 2011b; Li et al., 2013), quality traits (Lou et al., 2008; Zhao et al., 2008), and disease resistance traits (Piao et al., 2009; Chen et al., 2013), very few have been used for the genetic improvement of varieties in plant breeding programs. One important reason is that almost all QTL have been mapped in early segregating generations, which complicated the introgression of favorable QTL alleles. CSSLs provide a platform for accurate and advanced backcross QTL (AB-QTL) mapping that can be used for fine mapping of QTL as single Mendelian factors. To date, only few CSSL populations have been constructed and used for QTL analysis in *Brassica* crops, such as *B. napus* (Howell et al., 1996; Burns et al., 2003) and *B. oleracea* (Ramsay et al., 1996; Rae et al., 1999). In this study, we successfully developed a set of 63 CSSLs by using a marker-assisted backcrossing strategy for the introgression of chromosome segments of the donor parent “Chiifu” into the genetic background of “49caixin.” Each CSSL could be used as a starting material for developing near isolated lines by marker-assisted backcross, which can avoid unfavorable trait association referred to as “linkage drag” in breeding programs (Hospital, 2005; Yamamoto et al., 2009). Several elite advanced backcross lines that harbor resistance genes without linkage drag were developed in rice (Suh et al., 2011, 2013). Development and application of this set of CSSLs can accelerate this process in *B. rapa* crops.

Missing donor chromosome segments and residual non-targeted segments have been observed in many crops (Furuta et al., 2014). In our study, few gaps were found on chromosomes A01, A02, A05, A06, A07, and A09. These missing chromosome regions may contain genes involved in the reproductive barrier, thereby complicating their fixation in the genetic background of “49caixin.” In addition, some fragments between two linked markers were relatively long, such as on A01 between sau_um367 and BnGMS171 markers, on A04 between nia_060 and sau_um190 markers, on A05 between sau_um269 and cnu_029 markers, and on A09 between sau_um368 and nia_044 markers (Figure 5). Thus, these regions would need to be verified using additional markers to avoid missing unknown target segments. Recently, Xu et al. (2010) developed high-throughput genotyped CSSLs on the basis of whole-genome re-sequencing in rice; this method enabled the detection of numerous new segments compared to marker-based selection. This sequencing-based assisted approach could be used in *B. rapa* for CSSL construction to fill the gaps of donor chromosome segments and guarantee the accuracy of the substituted segments in our CSSLs. Among 63 CSSLs, 24 lines carried more than one segment of donor genome. It is necessary to purify them to single substituted segment lines by reducing the residual non-target fragments from the donor in further study.

Bolting and flowering times are two important agronomic traits that affect *B. rapa* crop production. Many studies have focused on bolting and flowering time QTL under different environmental conditions, by using primary mapping populations in *B. rapa*. For bolting time, *qDB-2-1, qDB-2-2, qDB-3-1, qDB-9-3*, and *qDB-10-2* have previously been identified (Li et al., 2009, 2013). Nine other QTL regions have been newly identified by this CSSL population for bolting time. For flowering time, *qDF-1-1* located on the top of A01 was in a similar region to *FLQTL-1* and *qFT1* identified by Lou et al. (2007) and Li et al. (2013), respectively. *qDF-2-1* identified on the top of A02 has also been detected in previous studies (Lou et al., 2007; Li et al., 2009, 2013). The *BrFLC2* gene located in this QTL region is a key gene that controls flowering time (Xiao et al., 2013). QTL *qDF-3-1, qDF-3-2, qDF-8-1, qDF-9-3, qDF-10-1*, and *qDF-10-2* have also been recently identified by Lou et al. (2007) and Li et al. (2013). Nine other QTL regions for this trait have been newly identified by this CSSL population. These new QTL present in the corresponding CSSLs provide a chance to isolate new candidate genes involved in bolting and flowering. In addition to genetic regulation, bolting, and flowering time depend on the growing season since they are affected by vernalization and photoperiod (Jack, 2004). Among the 14 DB and 17 DF QTLs, four DB and three DF QTL were detected in two seasons (August 2012 and January 2013), indicating that they are environment-independent loci. The other QTL were not detected in the winter season; thus, they are environment-sensitive loci. The lack of QTL detection could be attributed to the completion of vernalization or to the short-day conditions. Each CSSL that harbors these environment-sensitive QTL could be used to study the interaction between QTL and different environmental conditions.

Co-localization of QTL on chromosomes, referred to as “QTL clustering,” was observed for the phenotypic traits considered in this study, indicating that either multiple linkage loci/genes or pleiotropic loci may control these plant developmental traits. In this study, a few genomic regions that harbor QTL clusters were investigated, mainly those on chromosomes A01, A02, A08, A09, and A10. These co-localized QTL may explain the phenotypic correlation observed. QTL co-localization has also been widely reported for morphological and yield traits in *B. rapa* (Lou et al., 2007; Ge et al., 2011b; Li et al., 2013), *B. napus* (Quijada et al., 2006; Udall et al., 2006), and *B. juncea* (Ramchiary et al., 2007). Genomic regions at the top of A02 and bottom of A10 that affect flowering time, leaf traits, seed-related traits, and turnip formation were detected by both Lou et al. (2007) and Li et al. (2013); this indicated that these two regions may harbor important candidate genes for plant morphotype formation. However, in these studies, the QTL were located on relatively large genomic intervals, and precise mapping and gene isolation might be difficult because of the limitations regarding marker availability and population characteristics. In our results, these two regions, involved in DB, DF, PH, LW, and PD and FSD, were also identified and anchored to 1.4–4.2 Mb of A02 and 12.9–13.7 Mb of A10. They were carried by CSSLs SL05 and SL62, which would facilitate the development of secondary populations by using these two target CSSLs for further QTL validation. In addition, the genomic region on 0–1.8 Mb of the top of A09 containing a QTL cluster for DB, DF, PH, and PD has been newly identified. Pioneering studies on tomato and rice have provided good examples of map-based cloning of traits by using secondary CSSL-F_2_ populations (Frary et al., 2000; Yano et al., 2000; Chen et al., 2014). In the future, target CSSLs that harbor desirable QTL alleles such as SL05 and SL62 could be crossed to the recurrent parent “49caixin” to construct a secondary F_2_ population for QTL fine mapping. This could help in elucidating the genetic mechanism underlying co-located QTL, and, thus, accelerate genetic studies of complex morphological traits in *B. rapa*.

Compared to the model plant *Arabidopsis* and economical crops such as rice, the development of CSSLs in *B. rapa* has been relatively slow. In our study, we constructed a CSSL population by using two leafy-type *B. rapa* lines and verified the potential of this population for QTL detection of morphological traits. Nevertheless, the genetic detection of other traits such as heading, turnip shape, and oil content will require the development of additional CSSL populations by using a wide range of cross combinations. These CSSL populations could be used for mapping and cloning novel QTL/genes that govern corresponding desirable traits in *B. rapa* and would serve as rich plant materials for the *Brassica* research community.

In conclusion, we developed 63 CSSLs that cover nearly entire chromosome regions of the model *B. rapa* cultivar “Chiifu” on the genetic background of “49caixin,” which provided the opportunity to further explore the gene function and effects of “Chiifu” alleles. Our future research objectives will focus on the co-localized QTL regions on A02, A09, and A10, which have higher additive effects, in order to determine the genes that control different morphological traits. The identified QTL chromosomal regions present in the CSSLs could allow a regional candidate-gene association mapping approach in natural populations to reveal natural allelic variations that could explain the desirable traits. Moreover, this set of CSSLs will also allow us to proceed with field trials in different environments to identify novel QTL for other complex quantitative traits.

## Author contributions

XL analyzed and interpreted all data and drafted the manuscript. WW, ZW, and KL performed population development and phenotype measurement. YL participated in the data analysis and helped to draft the manuscript. ZP conceived the study, participated in its coordination, and helped to draft the manuscript. All authors have read and approved the final manuscript.

### Conflict of interest statement

The authors declare that the research was conducted in the absence of any commercial or financial relationships that could be construed as a potential conflict of interest.
